# Efficacy and Safety of Butorphanol Use in Patient-Controlled Analgesia: A Meta-Analysis

**DOI:** 10.1155/2021/5530441

**Published:** 2021-07-23

**Authors:** Zhihua Zhu, Wenyu Zhang

**Affiliations:** Department of Anesthesiology, China-Japan Union Hospital of Jilin University, Changchun, Jilin, China

## Abstract

**Objective:**

This meta-analysis evaluates the efficacy and safety regarding usage of butorphanol in patient-controlled analgesia (PCA).

**Methods:**

Several databases such as PubMed, Cochrane Library, Embase, CNKI, and VIP were explored with the help of computer search and manual retrieval. Randomized controlled trial (RCT) was selected, and the meta-analysis was conducted using RevMan 5.1. The primary efficacy endpoint was the postoperative visual analog scale score, postoperative Ramsay sedation scale (RSS), and adverse events.

**Results:**

Nine RCTs met the inclusion criteria and were included in this meta-analysis. No significant differences were found between the butorphanol group and nonbutorphanol group at 12 h and 48 h. The postoperative RSS score at 12 h, 24 h, and 48 h was representative of the calming effect of butorphanol. No significant difference was found on the endpoint of the postoperative RSS score at 12 h, 48 h, and 24 h. The RSS score was lower in the butorphanol group in comparison to the nonbutorphanol group. The butorphanol group was also associated with lower rate of nausea, vomiting, itching, and dizziness compared to the nonbutorphanol group.

**Conclusion:**

Butorphanol may be used in PCA as a successful postoperative analgesia and is also associated with lower side effects. Further research is needed to verify the efficacy and safety of butorphanol.

## 1. Introduction

Postoperative pain is a major problem. However, administering an effective analgesic helps in accelerating postoperative surgical recovery and rehabilitation [1]. Patient-controlled analgesia (PCA) is a method where patients can initiatively inject a dosage of medication, predetermined by their doctor, through a computer-controlled micropump press button.

Butorphanol is a synthetic opioid analgesic with an agonist activity for the *κ*-opioid receptor and antagonist activity for the *μ*-opioid receptor [2]. Butorphanol has analgesic effects similar to morphine but significantly lower adverse reactions such as respiratory inhibition, skin itching, and so on [3]. Therefore, in recent years, it has been widely used as an intraoperative adjuvant and postoperative PCA. Several studies have demonstrated that butorphanol is beneficial in treating postoperative pain after cesarean section, dental surgery, and pain experienced by patients suffering from migraine headache, acute musculoskeletal pain, and biliary colic [4–6]. However, there are no studies that analyze the use of butorphanol in PCA. This meta-analysis was undertaken to evaluate the efficacy and safety of butorphanol used in PCA.

## 2. Materials and Methods

This meta-analysis was conducted following the Preferred Reporting Items for Systematic Reviews and Meta-Analyses (PRISMA) [7] protocols for conducting a high-quality study.

### 2.1. Data Sources and Searches

Cochrane Library, EMBASE, PubMed, CNKI, and VIP databases were searched for RCTs, and the search time was set from January 1990 to April 2018. A sensitive filter was used for randomized control trials (RCTs), and the following keywords were used: “patient-controlled analgesia,” “butorphanol,” “analgesia,” and “postoperative pain.”

### 2.2. Study Selection

The inclusion criteria were as follows: (1) patients with postoperative pain; (2) butorphanol alone or in combination with other analgesics in the treatment group; (3) placebo or other analgesics except butorphanol in the control group; (4) the clinical outcomes of visual analog scale (VAS) score, Ramsay sedation scale (RSS) score, nausea, vomiting, itching, and dizzy were reported; (5) RCTs conducted in human beings. Studies were excluded if data were only available in the form of abstracts, conference proceedings, websites, or personal communication; case reports, case series, observational studies (e.g., case-control, cross-sectional, and cohort studies), systematic reviews and meta-analyses, letters to the editor, reviews, editorials, commentaries, studies on animal models, and basic science studies were also excluded. In case there were duplicate studies from the same trial, the latest reported data were included.

### 2.3. Data Extraction and Quality Assessment

Clinical data were independently extracted by two independent authors using the same extraction table. The third investigator was consulted to resolve any conflicting opinions. Authors' names, year of publication, and baseline characteristics of the participants were extracted from investigations included in the study. The following endpoints were also extracted: VAS score, RSS score, incidence of nausea, vomiting, itching, and dizziness. Additionally, information regarding blinding, random sequence generation, allocation concealment, indications for incomplete outcome data, indications for selective reporting, and other biases were also collected to evaluate the quality of the included investigations [8].

### 2.4. Statistical Analysis

Risk ratio (RR) and 95% confidence interval (CI) were used to report the differences in dichotomous data. Mean differences with 95% CI were used to report the differences in continuous outcomes. The Cochran *Q* test and *I*^2^ statistic were used to assess heterogeneity, and Cochran's *P* < 0.10 and *I*^2^ > 50 were considered to be indicative of significant heterogeneity. Pooled analyses were conducted using a fixed effect model, and if there was significant heterogeneity, a random effect model was used. Publication bias was assessed using Begg's test, and sensitivity analysis was conducted by excluding each individual study. Data analyses were performed by Review Manager (RevMan) software (Version 5.1, The Cochrane Collaboration, Copenhagen, Denmark) and STATA software (Version 11.1, Stata Corp LP, College Station, TX, USA).

## 3. Results

### 3.1. Search Results

**Figure 1:**
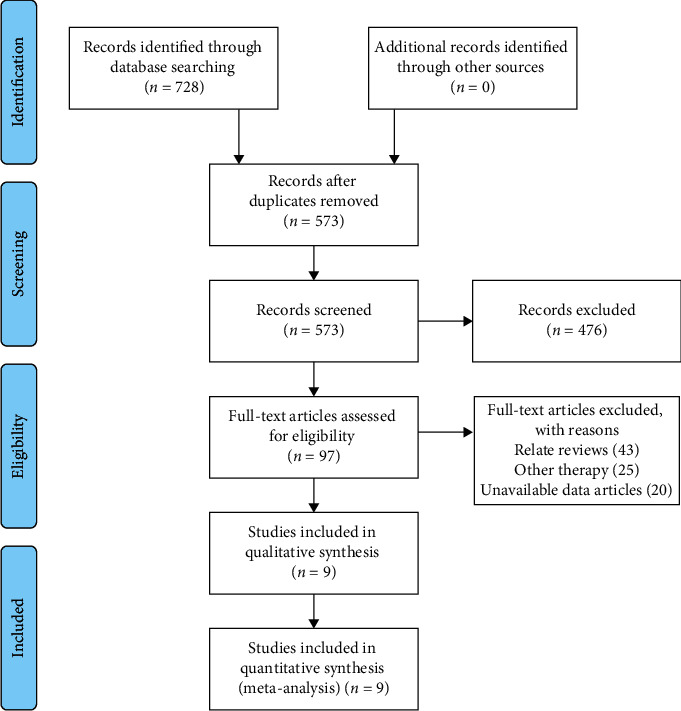
Flowchart showing the progress through the stages of the meta-analysis.

**Figure 2:**
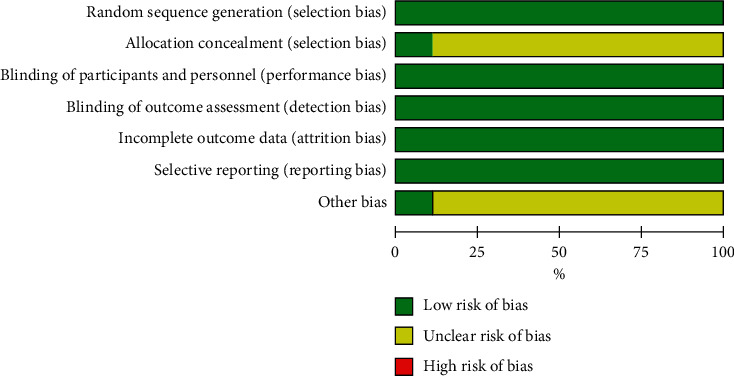
Risk of the bias graph.

**Figure 3:**
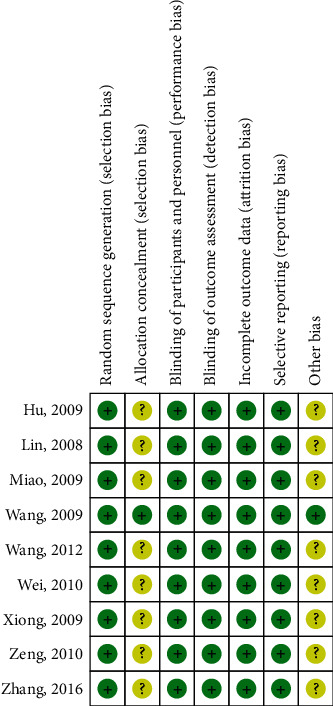
Risk of bias summary.

Of the 728 articles that were initially identified, nine clinical trials [9–17] satisfied the inclusion criteria of this study. The selection procedure is shown in Figure 1. In total, 426 patients were randomized to the butorphanol (experimental) group, and 420 patients were randomized to the nonbutorphanol (control) group. The baseline characteristics of the included studies are detailed in Table 1. The quality assessment is presented in Figures 2 and 3 . All clinical trials included in this study were characterized by a low risk of blinding of participants and outcome assessment, incomplete outcome data, and selective outcome reporting.

### 3.2. Clinical Results

The primary efficacy endpoint was VAS scores at 12 h, 24 h, and 48 h in the postoperative phase. Secondary endpoints included RSS scores at 12 h, 24 h, and 48 h in the postoperative phase. Safety endpoints were the rate of adverse events such as nausea, vomiting, itching, and dizziness.

### 3.3. VAS Score

**Figure 4:**
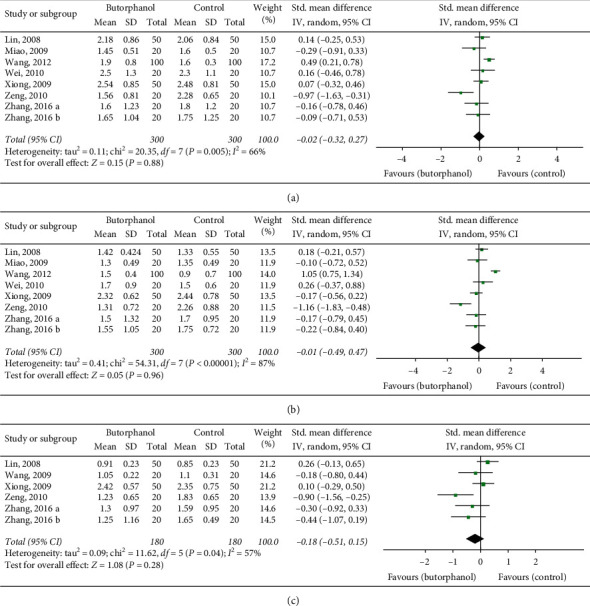
(a) Forest plot of 12 h postoperative VAS. (b) Forest plot of 24 h postoperative VAS. (c) Forest plot of 48 h postoperative VAS.

In this study, the postoperative 12 h, 24 h, and 48 h VAS scores were the primary endpoints, which are representatives of the effect of analgesic. There were no significant differences between the butorphanol group and nonbutorphanol group at 12 h (RR = −0.02; 95% CI = −0.32–0.27; *p*=0.88; *I*^2^ = 66%), 24 h (RR = −0.01; 95% CI = −0.49–0.47; *p*=0.96; *I*^2^ = 87%), and 48 h (RR = 0.18; 95% CI = −0.51–0.15; *p*=0.28; *I*^2^ = 57%) postoperative as shown in Figures 4(a)–4(c).

### 3.4. RSS Score

**Figure 5:**
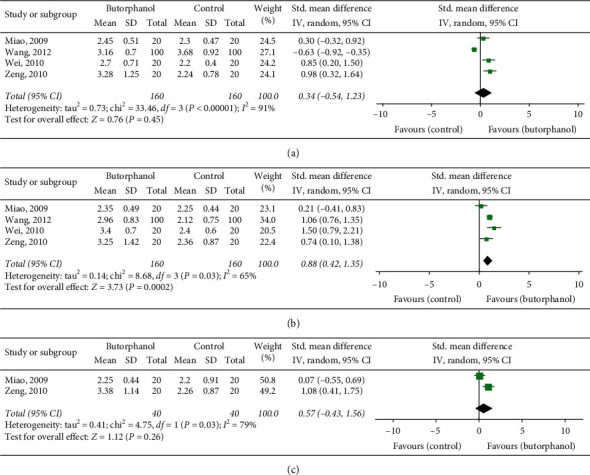
(a) Forest plot of 12 h postoperative RSS. (b) Forest plot of 24 h postoperative RSS. (c) Forest plot of 48 h postoperative RSS.

Postoperative RSS scores at 12 h, 24 h, and 48 h represented the calming effect. No significant difference was found on the endpoint of the postoperative RSS score at 12 h (RR = 0.34; 95% CI = –0.54–1.23; *p*=0.45; *I*^2^ = 91%) and 48 h (RR = 0.57; 95% CI = –0.43–1.56; *p*=0.26; *I*^2^ = 79%) as shown in Figures 5(a) and 5(b). However, the RSS score at 24 h was lower in the butorphanol group compared to the nonbutorphanol group (RR = 0.88; 95% CI = 0.42–0.53; *p*=0.0002; *I*^2^ = 65%) as shown in Figure 5(c).

### 3.5. Adverse Events

**Figure 6:**
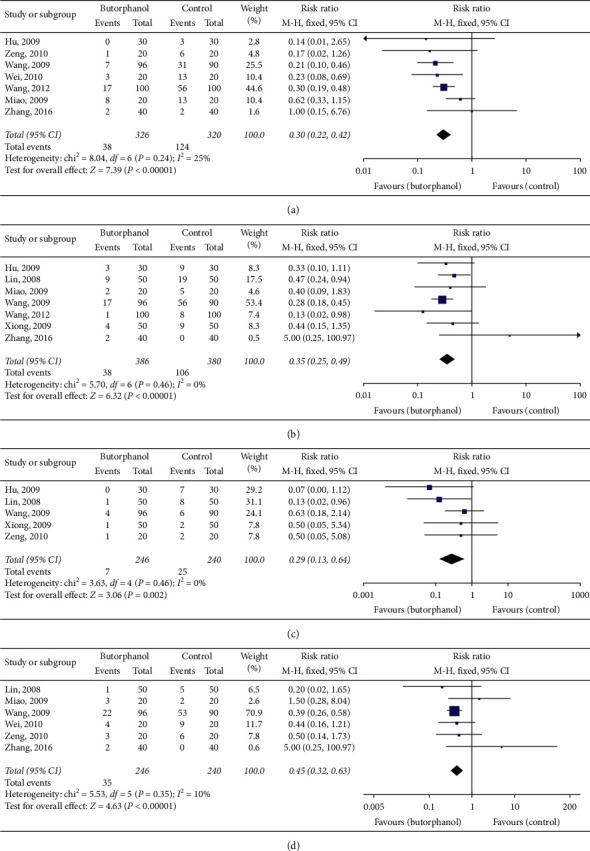
(a) Forest plot of nausea. (b) Forest plot of vomiting. (c) Forest plot of itching. (d) Forest plot of dizzy.

Common adverse events of butorphanol included nausea, vomiting, itching, and dizziness. The butorphanol group was associated with a lower rate of nausea (RR = 0.30; 95% CI = 0.22–0.42; *p* < 0.00001; *I*^2^ = 25%), vomiting (RR = 0.35; 95% CI = 0.25–0.49; *p* < 0.00001; *I*^2^ = 0), itching (RR = 0.29; 95% CI = 0.13–0.64; *p*=0.002; *I*^2^ = 0), and dizziness (RR = 0.45; 95% CI = 0.32–0.63; *p* < 0.00001; *I*^2^ = 10%) compared to the nonbutorphanol group as shown in Figure 6.

### 3.6. Sensitivity and Bias Analysis

**Figure 7:**
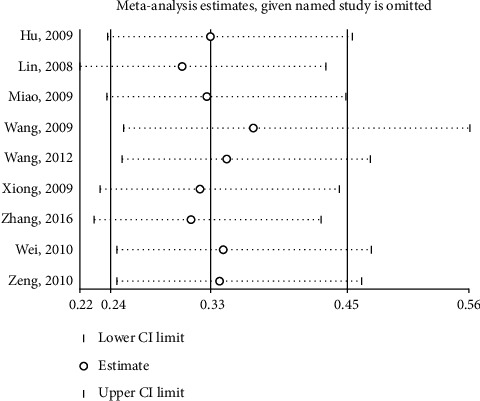
Sensitivity analysis of excluding each individual study.

Sensitivity analysis was conducted by excluding each individual study, and results obtained were similar to meta-analyses which demonstrated that the conclusion of this study was stable as shown in Figure 7. Begg's test and Egger's test for each study endpoints were conducted, and *p* values of each test are given in Table 2. These results indicate that there was a publication bias in VAS and RSS endpoints.

## 4. Discussion

This meta-analysis includes 426 patients randomized to a butorphanol group and a nonbutorphanol group within nine RCTs. Here, it was found that there are no significant differences between butorphanol and nonbutorphanol groups on the endpoint of postoperative 12 h, 24 h, and 48 h VAS scores and representative 12 h and 48 h RSS scores. However, the postoperative 24 h RSS score was reduced after administration of butorphanol. With regards to adverse events such as nausea, vomiting, itching, and dizziness, the rate of incidence was significantly reduced by butorphanol used.

Postoperative pain is a common complaint among patients after surgery. Apfelbaum et al. [18] reported that approximately 80% of the patients suffer postoperative pain and 86% of them experienced pain ranging from moderate to severe. Percutaneous pump methods such as PCA have been used to decrease postoperative pain, but with limited success. Butorphanol is commonly used for PCA in clinic. Palaacios et al. [19] and Pokharel et al. [20] reported about the clinical application of butorphanol in analgesia after cesarean section. Camann et al. [21] conducted a comparative study on intravenous analgesia of butorphanol after cesarean section in 1992 and highlighted the postoperative analgesic and sedative effect of butorphanol. Although there are many clinical studies which demonstrate the clinical use of butorphanol, this is the first meta-analysis that comprehensively evaluated the use of butorphanol in PCA.

There are several limitations in this study. First, the dosage of butorphanol and the surgery patients undertaken were slightly different in the clinical trials that were included in this meta-analysis. Second, other analgesics used in combination with butorphanol may contribute to differences in each study. Third, publication bias existed, the reason for which may be the limited number of studies were included. When the number of studies is less than 20, the sensitivity of Begg's and Egger's decreases. Finally, individual patient-level data could not be obtained to further analyze potential limitations. Overall, different design and characteristics of each trial might have caused heterogeneity. Given the important differences between trials, further randomized trials are warranted to facilitate a nuanced analysis.

## 5. Conclusion

In conclusion, butorphanol may be used in PCA as a successful postoperative analgesic and is also associated with limited side effects. Further experiments are needed to verify the efficacy and safety of butorphanol.

## Figures and Tables

**Table 1:** Baseline characters of included studies.

Study	Sample size		Analgesic methods		Population	Age		ASA physical status	
	T	C	T	C		T	C	T	C
Xiujuan et al., 2009 [16]	96	90	Butorphanol i.v. infusion with morphine PCA	Saline infusion with morphine PCA	Total abdominal hysterectomy	47 ± 15	46 ± 13	I/II	I/II
Zhang et al., 2016 a [17]	20	20	Butorphanol in combination with dexmedetomidine	Sufentanil in combination with dexmedetomidine	Laparoscopic resection of gastrointestinal tumors	55.00 ± 11.12	60.20 ± 9.27	I/II	I/II
Zhang et al., 2016 a [17]	20	20	Butorphanol in combination with dexmedetomidine	Sufentanil in combination with dexmedetomidine	Laparoscopic resection of gastrointestinal tumors	53.30 ± 10.42	55.50 ± 10.76	I/II	I/II
Hu et al., 2009 [9]	30	30	Butorphanol 4 mg + 1% ropivacaine 10 mL + saline diluted to 100 ml	Morphine 4 mg + 1% ropivacaine 10 mL + normal saline diluted to 100 mL	Hip replacement	73.8 ± 11.7	73.8 ± 11.7	I/II	I/II
Jianping and Zebin, 2012 [11]	100	100	Butorphanol tartrate 8 mg, fentanyl 1 mg plus 0.9% chlorination diluted to 150 ml	Fentanyl 1.5 mg, 0.9% sodium chloride diluted to 150 ml	Cesarean section	21–35	21–35	I/II	I/II
Huaping et al., 2009 [10]	50	50	Butorphanol 6 mg + fentanyl 0.6 mg + ondan diazem 8 mg, diluted to 100 mL with 0.9% saline	Fentanyl 1.2 mg + ondan diazem 8 mg diluted to 0.9 ml with 0.9% normal saline	Uterine and ovarian surgery	42 ± 10	40 ± 12	I/II	I/II
Xinxia et al., 2008 [15]	50	50	Butorphanol 5 mg + fentanyl 0.5 mg + 0.9% NaCl 100 mL	Fentanyl 1 mg+0.9% NaCl 100 ml	Subtotal hysterectomy	40–65	40–65	I/II	I/II
Wang et al. 2009 [13]	20	20	Butorphanol 7 mg + fentanyl 0.3 mg/100 ml	Fentanyl 1 mg/100 ml	Lower extremity fracture	18–50	18–50	I/II	I/II
Lai et al., 2010 [12]	20	20	Fentanyl 0.5 mg + butorphanol tartrate 5 mg + ondansetron 8 mg + normal saline to 100 mL	Fentanyl 1 mg + ondansetron 8 mg + saline to 100 mL	Patients under thyroid surgery	21–51	21–52	I/II	I/II
Xianyang et al., 2010 [14]	20	20	Butorphanol 5 mg + fentanyl 0.5 mg + ondansetron 8 mg	Fentanyl 1.0 mg + ondansetron 8 mg	Cesarean section	26.37 ± 2.37	26.13 ± 3.21		

T, treatment group; C, control group.

**Table 2:** Begg's test and Egger's test for each study endpoints.

Study endpoints	*P* value of Egger's test	*P* value of Begg's test
Nausea	0.958	0.851
Vomiting	0.599	0.881
Itching	0.224	0.142
Dizzy	0.333	0.188
12 h VAS	0.012	0.035
24 h VAS	0.026	0.216
48 h VAS	0.018	0.015
12 h RSS	0.025	0.042
24 h RSS	0.025	0.042

## Data Availability

The datasets used and/or analyzed to support the findings of this study are included within the article.

## References

[1] Kehlet H., Holte K. (2001). Effect of postoperative analgesia on surgical outcome. *British Journal of Anaesthesia*.

[2] Gillis J. C., Benfield P., Goa K. L. (1995). Transnasal butorphanol. *Drugs*.

[3] Commiskey S., Fan L.-W., Ho I. K., Rockhold R. W. (2005). Butorphanol: effects of a prototypical agonist-antagonist analgesic on *κ*-opioid receptors. *Journal of Pharmacological Sciences*.

[4] Dale O., Hjortkjaer R., Kharasch E. D. (2002). Nasal administration of opioids for pain management in adults. *Acta Anaesthesiologica Scandinavica*.

[5] Rapoport A. M., Bigal M. E., Tepper S. J., Sheftell F. D. (2004). Intranasal medications for the treatment of migraine and cluster headache. *CNS Drugs*.

[6] Wermeling D., Grant G., Lee A., Alexander N., Rudy A. (2005). Analgesic effects of intranasal butorphanol tartrate administered via a unit-dose device in the dental impaction pain model: a randomized, double-blind, placebo-controlled, parallel-group study. *Clinical Therapeutics*.

[7] Moher D., Liberati A., Tetzlaff J., Altman D. G. (2009). Preferred reporting items for systematic reviews and meta-analyses: the PRISMA statement. *Journal of Clinical Epidemiology*.

[8] Liu X.-Q., Luo X.-D., Wu Y.-Q. (2020). Efficacy and safety of bivalirudin vs heparin in patients with coronary heart disease undergoing percutaneous coronary intervention. *Medicine*.

[9] Hu D. H., Li Y. L., Cai M. X. (2009). Epidural butorphanol analgesia in elderly patients undergoing hip replacement. *Nan Fang Yi Ke Da Xue Xue Bao*.

[10] Huaping X., Zhiyong X., Aibing Z. (2009). Clinical observation of butorphanol combined with fentanyl in postoperative intravenous analgesia in gynecological patients. *An Hui Medical And Pharmaceutical Journal*.

[11] Jianping W., Zebin L. (2012). Clinical observation of butorphanol combined with fentanyl in postpartum analgesia. *Modern Medicine & Health*.

[12] Lai W., Jun-mei X., Zi-liang Q. (2010). Postoperative patient-controlled intravenous analgesia with butorphanol and fentanyl after thyroidectomy. *China Journal Of Modern Medicine*.

[13] Wang F., Shen X., Liu Y., Xu S., Guo X. (2009). Continuous infusion of butorphanol combined with intravenous morphine patient-controlled analgesia after total abdominal hysterectomy: a randomized, double-blind controlled trial. *European Journal of Anaesthesiology*.

[14] Xianyang Z., Jize L., Guiqin L. (2010). Combined buprenorphine with fentanil for patient-control intravenous analgesia after cesarean section. *Medical Journal Of Liaoning*.

[15] Xinxia L., Xuebin J., Qianhuang C., Yi J., Guifan Z. (2008). Butorphanol combined with fentanyl for intravenous analgesia after gynecological surgery. *Zhe Jiang Journal Of Integrated Traditional Chinese And Western Medicine*.

[16] Xiujuan M., Ruihua P., Xinfa N., Tao L., Laiyou W. (2009). Analysis of the efficacy of butorphanol in operational analgesia of lower limb fractures. *Chinese Journal Of Misdiagnostics*.

[17] Zhang X.-K., Chen Q.-H., Wang W.-X., Hu Q. (2016). Evaluation of dexmedetomidine in combination with sufentanil or butorphanol for postoperative analgesia in patients undergoing laparoscopic resection of gastrointestinal tumors. *Medicine*.

[18] Apfelbaum J. L., Chen C., Mehta S. S., Gan A. T. J. (2003). Postoperative pain experience: results from a national survey suggest postoperative pain continues to be undermanaged. *Anesthesia & Analgesia*.

[19] Palacios Q. T., Jones M. M., Hawkins J. L. (1991). Post-caesarean section analgesia: a comparison of epidural butorphanol and morphine. *Canadian Journal of Anaesthesia*.

[20] Pokharel K., Rahman T. R., Singh S. N., Bhattarai B., Basnet N., Khaniya S. (2008). The efficacy and safety of low dose epidural butorphanol on postoperative analgesia following cesarean delivery. *NMA: Journal of the Nepal Medical Association*.

[21] Camann W. R., Loferski B. L., Fanciullo G. J., Stone M. L., Datta S. (1992). Does epidural administration of butorphanol offer any clinical advantage over the intravenous route? a double-blind, placebo-controlled trial. *Anesthesiology*.

