# Corrigendum to “Efficacy and Safety of Butorphanol Use in Patient-Controlled Analgesia: A Meta-Analysis”

**DOI:** 10.1155/2021/9854850

**Published:** 2021-10-23

**Authors:** Zhihua Zhu, Wenyu Zhang

**Affiliations:** Department of Anesthesiology, China-Japan Union Hospital of Jilin University, Changchun, Jilin, China

In the article titled “Efficacy and Safety of Butorphanol Use in Patient-Controlled Analgesia: A Meta-Analysis” [1], the funding statement was omitted by mistake and is as follows.
